# Meetings of Societies

**Published:** 1925

**Authors:** 


### MEETINGS OF SOCIETIES.

### Bristol Medico-Chirurgical Society.

*February 11th, 1925.*

In the absence of the President, Mr. J. Lacy Firth took the chair.

Mr. Percy Sargent, C.M.G., D.S.O., M.S., read a paper on "**The Present Position of Surgery of the Nervous System,**" which appears in this issue (page 101). A vote of thanks was proposed to Mr. Sargent for the very interesting paper, and especially for the lantern slides which had illustrated it.

Mr. A. W. Adams briefly summarised notes of a case of "**Spinal Tumour**" which he had shown at the beginning of the meeting.

---

*March 11th, 1925.*

The President, Dr. J. O. Symes, in the Chair.

Dr. James Swain, C.B., C.B.E., M.S., was elected an Honorary Member of the Society. Dr. Swain had been for twenty-five years a member of the Editorial Committee of the Society's *Journal,* and for many years longer a very active member of the Society. The President, in announcing the result of the vote, pointed out that Honorary Membership was very rarely conferred, there being only two other living Honorary Members.

Mr. H. Chitty, M.S., F.R.C.S., showed three cases :—

1. Osteomalacia in a girl of 18. Union of ununited fractures of the tibiæ followed local injections of colloidal magnesium.

2. Ununited fracture of the tibia in a child of 5. Local injections of magnesium and a rib graft had alike proved ineffectual.

3. Double pleural empyema in a child. Both sides of the chest were opened at the same operation, with perfect result.

Mr. A. W. Adams, F.R.C.S., showed a case of "**Malignant Disease of the Thyroid, with Secondary Glands, relieved by Operation.**" Specimens and sections were shown with the case.

Dr. R. C. Clarke, O.B.E., M.R.C.P., read a paper on "**The Catarrhal Child,**" which is to be published in another journal.

*Wednesday, April 8th.*

Dr. J. O. Symes, President, in the Chair.

Dr. Patrick Watson-Williams read a paper on "**Some Points in the Differential Diagnosis of Focal Infection in the Nasal Sinuses causing Ocular and other Systemic Infections,**" illustrated by lantern slides.

Mr. A. Rendle Short read a paper on "**Diagnosis of the Dyspepsias.**"

Both of these papers are published in this issue (*vide* pp. 88 & 121).

